# Eco-friendly approach for biosorption of Pb^2+^ and carcinogenic Congo red dye from binary solution onto sustainable *Ulva lactuca* biomass

**DOI:** 10.1038/s41598-020-73031-1

**Published:** 2020-09-29

**Authors:** Noura El-Ahmady El-Naggar, Nashwa H. Rabei, Sahar E. El-Malkey

**Affiliations:** 1 Department of Bioprocess Development, Genetic Engineering and Biotechnology Research Institute, City of Scientific Research and Technological Applications (SRTA-City), Alexandria, 21934 Egypt; 2 Microbial Biotechnology Department, Genetic Engineering and Biotechnology Research Institute, University of Sadat City, Sadat City, Egypt

**Keywords:** Environmental biotechnology, Biochemistry, Environmental sciences

## Abstract

Dyes constitute an important group of organic contaminants and are recognized for its harmful effects on the aquatic environments and humans. Heavy metals are also the largest group of inorganic pollutants due to their accumulation in the environment, contaminate food chains and cause adverse effects on the living organisms. Biosorption capacity of *Ulva lactuca* biomass was assessed in batch experiments for simultaneous removal of Pb^2+^ and Congo red dye from binary solution. The process variables effects on Congo red dye and Pb^2+^ removal percentages were explored by performing 50 experiments using Face-centered central composite design. The highest removal percentages of Congo red dye (97.89%) and Pb^2+^ (98.78%) were achieved in the run no. 24, using 100 mg/L Congo red dye, 200 mg/L Pb^2+^, 3 g/L algal biomass, initial pH 6 and contact time was 120 min at 30 °C. FTIR analysis of the algal biomass showed the existence of many functional groups responsible for the biosorption process. After the biosorption process, SEM analysis revealed obvious morphological changes including surface shrinkage and the presence of new glossy Pb^2+^ particles, and the EDS spectra reveals presence of additional Pb^2+^ peak confirming the capacity of *Ulva lactuca* biomass to remove Pb^2+^ from binary solution.

## Introduction

Several studies have been carried out on single-pollutant adsorption, while substantial amounts of both dyes and metals are found together in the industrial wastewater^1^. Thus, a lot of attention was paid to the simultaneous removal of many coexisting mixed pollutants as dyes and heavy metal ions in the processes of wastewater treatment^2^. Dyes are the main industrial coloring substances used extensively in the leather, textile, paper, fruit, cosmetics and pharmaceutical industries. It was estimated that approximately 12% of the synthetic colorants are lost during the dyeing process and approximately 20% of these lost colorants released into the industrial wastewaters^3^. Globally, more than 800,000 tons of synthetic dyes are discharged annually in the aquatic environments^4^. The discharge of dyes from various industries into water rivers and lakes and surrounding industrial areas even at a very small concentration of less than 1 ppm are highly toxic, leads to a reduction in dissolved oxygen concentration which subsequently affect aerobic organisms and causing several adverse effects on aquatic ecosystem and may be carcinogenic^5, 6^. Small amounts of dyes present high effects both on the color and water quality^7^.

**Figure 1:**
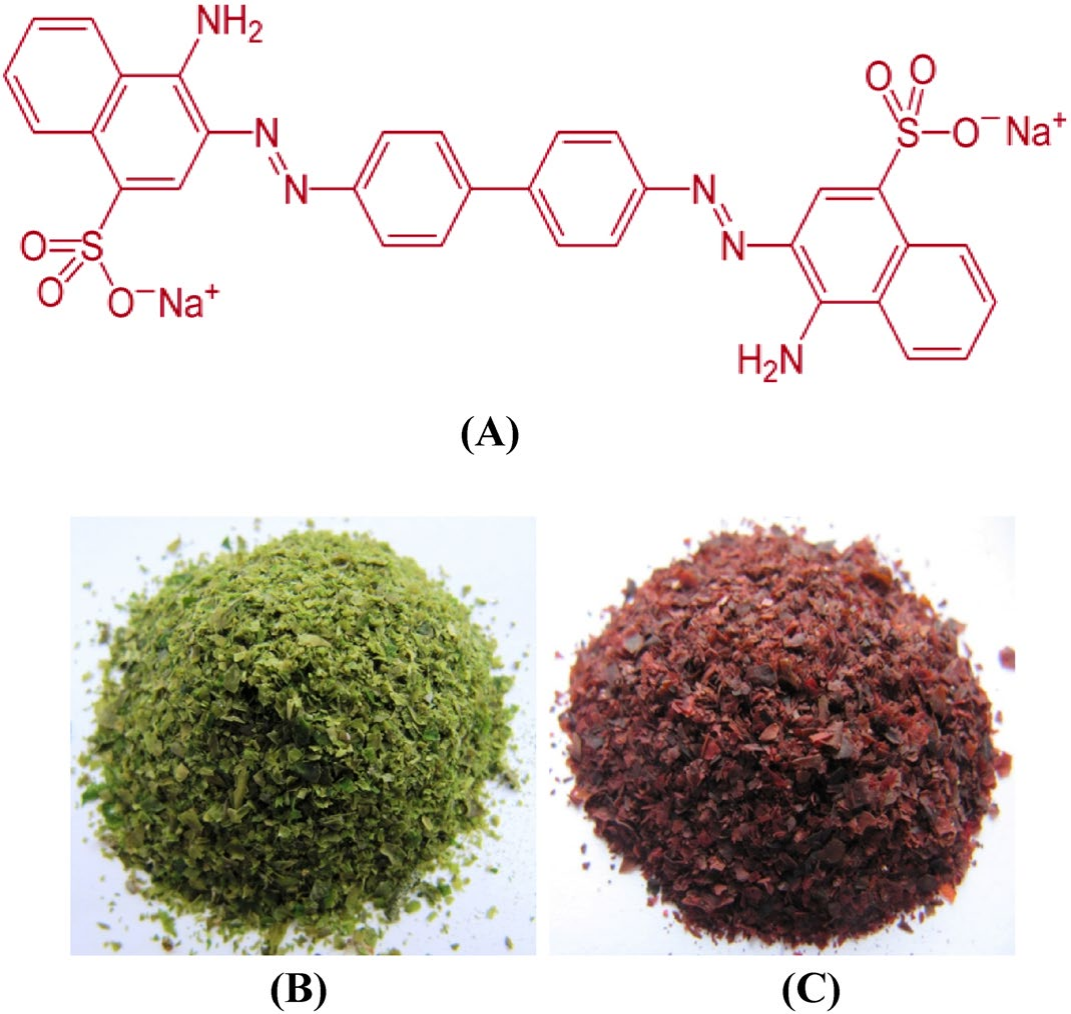
(**A**) Formula of Congo red dye. The dried, milled biomass of *Ulva lactuca* before (**B**) and after (**C**) simultaneous biosorption of Congo red dye and Pb^2+^.

Congo red (Fig. 1A) is a benzidine-based anionic diazo dye with color-assisting groups (amino and sodium sulfonate). It widely used for dyeing wool, silk, paper, leather, jute, cotton and plastic industries^8^ because of its low cost. This dye can be discharged with sewage and poses potential health, environmental and ecological problems^9^ because of poor degradation. Congo red affects blood factors including clotting and acts as eye, skin and gastrointestinal irritant. It causes allergic reactions, drowsiness and breathing difficulties^10^. Congo red known to metabolize to benzidine—a human mutagenic and carcinogenic product^11, 12^. In the presence of extensive coloration of Congo red in the aqueous environments, reduce the penetration of sunlight into deep layers that impair photosynthetic function, deteriorate water quality and also decrease gasses solubilities, causing acute toxic effects on aquatic fauna and flora^13, 14^. Due to the adverse effects of Congo red on the environment and human health, its elimination is nowadays one of the growing demands.

Heavy metals also constitute the largest group of inorganic contaminants in the environment. A large number of industries including fertilizers, pesticides, mining, pigments, battery manufacturing, petroleum refining and plating release heavy metals into waste streams that adversely affect water bodies^15^. Lead (Pb^2+^) is one of the most abundant toxic heavy metals affecting the environment. It has been discharged into the environment through disposal of municipal sewage sludge enriched with lead, automobile batteries manufacture, textile dyeing, paper and printing processes, pulp, photographic and paint materials, petroleum refining, glass and ceramic industries, and mining processes^16^. Lead in the environment even at low concentrations poses a serious threat to living organisms, including animals and humans. Lead accumulates mainly in muscles, kidney, brain and bones^17^. Acute exposure to lead induces severe damage to the kidneys and brain, high exposure to lead may reduce fertility in males and cause pregnant women miscarriage^18^. Lead toxicity was found to cause anemia as lead inhibits or induces inefficient synthesis of the heme^19^. Chronic lead poisoning causes hypertension and cardiovascular disease^20^, liver diseases, gastrointestinal damage, abdominal pain, renal failure, loss of coordination, motor impairment, paralysis, weakness of the body, nervous disorders, loss of short-term memory, nausea, depression and mental retardation in children^21, 22^. Additionally, Pb^2+^ interferes with calcium in the body that disrupts the release of neurotransmitter and the mineral density of bones^23^. It is therefore, essential to remove Pb^2+^ from industrial wastes before disposal into water streams.

Several physico-chemical approaches have been widely used for Azo dyes removal from wastewater including ozonation, photocatalytic degradation, chemical degradation, coagulation, neutralization, lime softening, precipitation, membrane filtration, ion exchange, adsorption and oxidation process^24^. These physico-chemical approaches have been restricted due to a high cost, generation of toxic sludge after removal and restricted application in most cases^25^. Additionally, several physico-chemical strategies have been widely used for removing metal ions from wastewater including ion exchange, reverse osmosis, evaporation, electrochemical treatment, oxidation or reduction, chemical precipitation and filtration^26^. However, these strategies were limited due to high reagents or energy requirements, costly equipment and monitoring systems, incomplete metal removal and/or toxic sludge generation or other toxic substances requiring an appropriate disposal process. Therefore, there is an urgent need for save and inexpensive alternative method. Consequently, biological treatment (biosorption) is a promising, eco-friendly, cost effective and efficient alternative technique to the traditional treatment technologies for treatment of contaminated wastewater^27–29^ to remove textile dyes and heavy metals. It has been documented that yeast, bacteria, algae, filamentous fungi and their enzymes have been successfully used for removal of hazardous azo dyes^30^. Algae are considered to be effective sustainable biosorbents for metal removal owing to its high metal-binding affinity, low-cost and local abundance in both fresh and salt water and surface characteristics^31–33^. Dulla et al.^34^ revealed that the high removal and biosorption capacity of algae was mainly attributed to their surface functional groups like hydroxyl, amino and carboxylic, which binds with metal ions through various mechanisms such as electrostatic forces, ion exchange or complexation.

*Ulva lactuca* is sustainable biomass feedstocks for bioremediation, green source for production of food, biofuels, bioactive compounds, can be a source of essential amino acids and production of high value products^35, 36^.

Optimization of the operating process variables of the biosorption process is of great importance in the development of the process. Response surface methodology (RSM) was applied for several decades for optimization of different process variables. RSM is mathematical and statistical techniques commonly used for understanding the interactions between the different process factors and to calculate the optimal levels of the variables significantly affecting the response^37, 38^.

The main goal of this study was to investigate simultaneous biosorption of Pb^2+^ and a carcinogenic Congo red dye from binary solution using the biomass of marine algae, *Ulva lactuca*, as a cost effective biosorbent. Response surface methodology has been used to optimise the operating parameters of the biosorption process. Characterization of the *Ulva lactuca* biomass before and after biosorption process also performed using FTIR spectra analysis, Scanning Electron Microscopy (SEM) and Energy-dispersive spectroscopy (EDS) analysis.

## Results and discussion

### Statistical optimization of simultaneous bioremoval of Pb^2+^ and Congo red dye from binary solution by ***Ulva lactuca*** biomass.

Biosorption processes are affected by many environmental factors such as initial pH level, biosorbent dosage, contact time, temperature and initial metal concentration^39^. Optimization of biosorption process parameters was carried out using the Face-centered central composite design (FCCCD) to maximize the removal percentages and to study the individual, interaction and quadratic effects of process variables on simultaneous bioremoval of Pb^2+^ and Congo red dye from binary solution by dry *Ulva lactuca* biomass. FCCCD was also used to predict the best biosorption conditions of the simultaneous bioremoval of Pb^2+^ and Congo red dye. To optimize the selected variables (*Ulva lactuca* biomass concentration, initial pH level, Pb^2+^ concentration, Congo red dye concentration and contact time), the FCCCD of 50 experimental runs was used. Table 1 presents the FCCCD which had 10 axial points, 32 factorial and 8 replicates at the centre points, the actual and coded levels of the five variables, the experimental and predicted removal percentages of Pb^2+^ and Congo red dye and the residuals. The obtained results of FCCCD experiments for simultaneous bioremoval of Pb^2+^ and Congo red dye show considerable variations in the removal percentage of Pb^2+^ and Congo red dye. Removal percentage of Pb^2+^ ranged from 78.23 to 98.78% and removal percentage of Congo red dye ranged from 13.16 to 97.89%.

**Table 1:** FCCCD matrix mean actual and predicted values of simultaneous biosorption of Congo red and Pb^2+^ by using *Ulva lactuca* biomass.

Std	Run	Type	X_1_	X_2_	X_3_	X_4_	X_5_	Congo red removal (%)			Pb^2+^ removal (%)		
								Actual	Predicted	Residuals	Actual	Predicted	Residuals
42	1	Axial	0	0	0	0	1	56.12	57.15	− 1.03	96.11	96.18	− 0.07
32	2	Fact	1	1	1	1	1	45.29	46.01	− 0.72	96.90	96.70	0.20
50	3	Center	0	0	0	0	0	94.76	93.67	1.09	98.15	96.49	1.66
26	4	Fact	1	− 1	− 1	1	1	41.36	40.12	1.24	90.81	90.32	0.49
46	5	Center	0	0	0	0	0	93.70	93.67	0.03	96.16	96.49	− 0.33
15	6	Fact	− 1	1	1	1	− 1	73.08	73.81	− 0.73	95.73	97.57	− 1.84
22	7	Fact	1	− 1	1	− 1	1	55.24	55.21	0.03	86.04	86.01	0.03
13	8	Fact	− 1	− 1	1	1	− 1	62.91	61.90	1.01	93.53	92.79	0.74
30	9	Fact	1	− 1	1	1	1	59.93	57.93	2.00	89.29	89.38	− 0.09
28	10	Fact	1	1	− 1	1	1	13.16	14.40	− 1.24	96.17	97.39	− 1.22
7	11	Fact	− 1	1	1	− 1	− 1	83.87	85.02	− 1.15	90.39	89.55	0.84
19	12	Fact	− 1	1	− 1	− 1	1	60.76	58.87	1.89	86.63	86.66	− 0.03
16	13	Fact	1	1	1	1	− 1	65.37	64.98	0.39	96.13	95.74	0.39
3	14	Fact	− 1	1	− 1	− 1	− 1	77.40	79.33	− 1.93	85.33	85.85	− 0.52
27	15	Fact	− 1	1	− 1	1	1	15.34	14.69	0.65	89.56	89.45	0.11
20	16	Fact	1	1	− 1	− 1	1	45.14	45.93	− 0.79	94.75	94.34	0.41
34	17	Axial	1	0	0	0	0	91.14	93.48	− 2.34	97.97	97.70	0.27
29	18	Fact	− 1	− 1	1	1	1	51.35	52.31	− 0.96	92.00	92.26	− 0.26
12	19	Fact	1	1	− 1	1	− 1	48.01	45.92	2.09	93.76	93.25	0.51
11	20	Fact	− 1	1	− 1	1	− 1	50.06	50.00	0.06	91.57	91.14	0.43
18	21	Fact	1	− 1	− 1	− 1	1	56.87	55.52	1.35	90.13	89.69	0.44
48	22	Center	0	0	0	0	0	93.35	93.67	− 0.32	96.57	96.49	0.08
17	23	Fact	− 1	− 1	− 1	− 1	1	56.83	57.79	− 0.96	88.72	88.89	− 0.17
33	24	Axial	− 1	0	0	0	0	97.89	99.03	− 1.14	98.78	98.21	0.57
6	25	Fact	1	− 1	1	− 1	− 1	45.63	46.17	− 0.54	78.23	78.22	0.01
21	26	Fact	− 1	− 1	1	− 1	1	60.87	62.23	− 1.36	89.15	89.15	0.00
24	27	Fact	1	1	1	− 1	1	58.38	59.42	− 1.04	90.75	90.91	− 0.16
9	28	Fact	− 1	− 1	− 1	1	− 1	52.88	51.90	0.98	86.35	86.62	− 0.27
49	29	Center	0	0	0	0	0	96.83	93.67	3.16	94.87	96.49	− 1.62
8	30	Fact	1	1	1	− 1	− 1	65.48	63.54	1.94	87.38	87.46	− 0.08
35	31	Axial	0	− 1	0	0	0	89.72	90.66	− 0.94	92.59	92.40	0.19
40	32	Axial	0	0	0	1	0	80.90	82.01	− 1.11	96.96	96.37	0.59
5	33	Fact	− 1	− 1	1	− 1	− 1	58.16	56.98	1.18	87.11	87.19	− 0.08
41	34	Axial	0	0	0	0	− 1	67.84	70.28	− 2.44	94.04	93.13	0.91
47	35	Center	0	0	0	0	0	96.05	93.67	2.38	94.59	96.49	− 1.90
37	36	Axial	0	0	− 1	0	0	81.62	84.16	− 2.54	93.80	92.81	0.99
36	37	Axial	0	1	0	0	0	89.28	91.82	− 2.54	97.76	97.11	0.65
14	38	Fact	1	− 1	1	1	− 1	62.45	63.74	− 1.29	83.93	84.09	− 0.16
39	39	Axial	0	0	0	− 1	0	92.95	95.31	− 2.36	92.29	92.04	0.25
10	40	Fact	1	− 1	− 1	1	− 1	56.88	58.49	− 1.61	81.51	81.85	− 0.34
31	41	Fact	− 1	1	1	1	1	51.50	51.05	0.45	93.70	92.70	1.00
43	42	Center	0	0	0	0	0	95.31	93.67	1.64	96.67	96.49	0.18
38	43	Axial	0	0	1	0	0	94.97	95.91	− 0.94	94.04	94.19	− 0.15
23	44	Fact	− 1	1	1	− 1	1	78.84	77.12	1.72	86.77	87.18	− 0.41
1	45	Fact	− 1	− 1	− 1	− 1	− 1	66.59	65.10	1.49	83.90	83.74	0.16
45	46	Center	0	0	0	0	0	96.07	93.67	2.40	93.87	96.49	− 2.62
25	47	Fact	− 1	− 1	− 1	1	1	28.53	29.74	− 1.21	88.97	89.26	− 0.29
2	48	Fact	1	− 1	− 1	− 1	− 1	58.64	59.04	− 0.40	78.32	78.72	− 0.40
4	49	Fact	1	1	− 1	− 1	− 1	63.55	62.61	0.94	87.39	87.70	− 0.31
44	50	Center	0	0	0	0	0	97.19	93.67	3.52	97.67	96.49	1.18
Variable					Variable code			− 1	0	1			
Congo red concentration (mg/L)					X_1_			100	150	200			
Pb^2+^ concentration (mg/L)					X_2_			100	200	300			
Algal biomass (g/L)					X_3_			1	3	5			
Initial pH level					X_4_			4	6	8			
Contact time (min)					X_5_			60	120	180			

The highest removal % of both Pb^2+^ and Congo red dye were achieved in the run no. 24 with percent of 98.78% and 97.89% for Pb^2+^ and Congo red dye; respectively, where Congo red dye concentration was 100 mg/L, Pb^2+^ concentration was 200 mg/L, by using 3 g/L algal biomass, initial pH 6 and 120 min of contact time at 30 °C. On the other hand, the minimum removal percentages of Pb^2+^ and Congo red dye were obtained in run numbers 25, 10; respectively. Figure 1B,C showed the dried, milled biomass of *Ulva lactuca* before (B) and after (C) of Congo red dye and Pb^2+^ simultaneous biosorption.

### Statistical analysis using multiple regression analysis and ANOVA

Both Pb^2+^ and Congo red dye removal (%) data were statistically analyzed by using multiple regression analysis and the results were presented in Tables 2, 3, 4, 5. The analysis includes the coefficient values, coefficient of determination (R^2^) which determines the efficiency of the polynomial regression model, the predicted R^2^ value, the adjusted R^2^ value, the effect of each variable, Fisher test (*F*-test) and probability *P-*value. Linear, interactions and quadratic effects of the selected five process parameters were also estimated.

**Table 2:** Analysis of variance for biosorption of Congo red by *Ulva lactuca* biomass obtained by FCCCD.

Source of variance		Degrees of freedom	Sum of square	Mean of square	*F*-value	*P*-value	Coefficient estimate
Model		20	22,728.56	1136.43	271.25	< 0.0001*	93.67
Linear effect	X_1_	1	261.77	261.77	62.48	< 0.0001*	− 2.77
	X_2_	1	11.38	11.38	2.72	0.1101	0.58
	X_3_	1	1172.94	1172.94	279.96	< 0.0001*	5.87
	X_4_	1	1504.90	1504.90	359.19	< 0.0001*	− 6.65
	X_5_	1	1466.42	1466.42	350.01	< 0.0001*	− 6.57
Interaction effect	X_1_X_2_	1	227.64	227.64	54.33	< 0.0001*	− 2.67
	X_1_X_3_	1	45.20	45.20	10.79	0.0027*	− 1.19
	X_1_X_4_	1	319.98	319.98	76.37	< 0.0001*	3.16
	X_1_X_5_	1	28.67	28.67	6.84	0.0140*	0.95
	X_2_X_3_	1	381.09	381.09	90.96	< 0.0001*	3.45
	X_2_X_4_	1	520.60	520.60	124.26	< 0.0001*	− 4.03
	X_2_X_5_	1	346.17	346.17	82.63	< 0.0001*	− 3.29
	X_3_X_4_	1	656.76	656.76	156.76	< 0.0001*	4.53
	X_3_X_5_	1	315.44	315.44	75.29	< 0.0001*	3.14
	X_4_X_5_	1	440.97	440.97	105.25	< 0.0001*	− 3.71
Quadratic effect	X_1_^2^	1	16.47	16.47	3.93	0.0569	2.58
	X_2_^2^	1	14.66	14.66	3.50	0.0716	− 2.43
	X_3_^2^	1	32.76	32.76	7.82	0.0091*	− 3.64
	X_4_^2^	1	62.06	62.06	14.81	0.0006*	− 5.01
	X_5_^2^	1	2219.22	2219.22	529.69	< 0.0001*	− 29.95
Error effect	Lack of Fit	22	107.87	4.90	2.52	0.1062	
	Pure Error	7	13.63	1.95			
R^2^	0.9947	Std. Dev	2.05				
Adj R^2^	0.9910	Mean	67.52				
Pred R^2^	0.9843	C.V. %	3.03				
Adeq Precision	63.80	PRESS	358.04				

“* Significant values, *F*: Fishers's function, *P*: Level of significance, C.V: Coefficient of variation”.

**Table 3:** Analysis of variance for biosorption of Pb^2+^ by *Ulva lactuca* biomass obtained by FCCCD.

Source of variance		Degrees of freedom	Sum of square	Mean of square	*F*-value	*P*-value	Coefficient estimate
Model		20	1263.97	63.20	61.08	< 0.0001*	96.49
Linear effect	X_1_	1	2.24	2.24	2.17	0.1518	− 0.26
	X_2_	1	188.66	188.66	182.32	< 0.0001*	2.36
	X_3_	1	16.10	16.10	15.56	0.0005*	0.69
	X_4_	1	159.28	159.28	153.93	< 0.0001*	2.16
	X_5_	1	79.07	79.07	76.42	< 0.0001*	1.53
Interaction effect	X_1_X_2_	1	94.60	94.60	91.42	< 0.0001*	1.72
	X_1_X_3_	1	31.09	31.09	30.04	< 0.0001*	− 0.99
	X_1_X_4_	1	0.14	0.14	0.13	0.7178	0.07
	X_1_X_5_	1	67.86	67.86	65.58	< 0.0001*	1.46
	X_2_X_3_	1	0.13	0.13	0.12	0.7281	0.06
	X_2_X_4_	1	11.69	11.69	11.30	0.0022*	0.60
	X_2_X_5_	1	37.58	37.58	36.32	< 0.0001	− 1.08
	X_3_X_4_	1	14.93	14.93	14.43	0.0007*	0.68
	X_3_X_5_	1	20.22	20.22	19.55	0.0001*	− 0.80
	X_4_X_5_	1	12.50	12.50	12.08	0.0016*	− 0.63
Quadratic effect	X_1_^2^	1	5.32	5.32	5.14	0.0310*	1.47
	X_2_^2^	1	7.43	7.43	7.18	0.0120*	− 1.73
	X_3_^2^	1	22.09	22.09	21.35	< 0.0001*	− 2.99
	X_4_^2^	1	12.90	12.90	12.46	0.0014*	− 2.28
	X_5_^2^	1	8.31	8.31	8.03	0.0083*	− 1.83
Error effect	Lack of Fit	22	14.03	0.64	0.28	0.9899	
	Pure Error	7	15.97	2.28			
R^2^	0.9768	Std. Dev	1.02				
Adj R^2^	0.9608	Mean	91.48				
Pred R^2^	0.9474	C.V. %	1.11				
Adeq Precision	30.32	PRESS	68.01				

* Significant values, *F*: Fishers's function, *P*: Level of significance, C.V: Coefficient of variation.

**Table 4:** Fit summary for FCCCD for adsorption of Congo red results.

Lack of fit tests					
Source	Sum of Squares	*df*	Mean Square	*F-*value	*P-*value *P*rob > *F*
Linear	18,419.02	37	497.81	255.66	< 0.0001*
2FI	15,136.50	27	560.61	287.91	< 0.0001*
Quadratic	107.87	22	4.90	2.52	0.1062

Sequential model sum of squares					
Source	Sum of Squares	*df*	Mean Square	*F-*value	*P-*value *P*rob > *F*
Linear vs Mean	4417.41	5	883.48	2.11	0.0822
2FI vs Linear	3282.53	10	328.25	0.74	0.6854
Quadratic vs 2FI	15,028.63	5	3005.73	717.42	< 0.0001*

Model Summary Statistics					
Source	Standard deviation	R-Squared	Adjusted R-Squared	Predicted R-Squared	PRESS
Linear	20.47	0.19332	0.1017	0.0066	22,698.94
2FI	21.11	0.33698	0.0445	− 0.2600	28,790.69
Quadratic	2.05	0.99468	0.9910	0.9843	358.04

“* Significant values, *df* : degree of freedom, PRESS: sum of squares of prediction error, two factors interaction: 2FI”.

**Table 5:** Fit summary for FCCCD for biosorption of Pb^2+^ results.

Lack of Fit Tests					
Source	Sum of Squares	*df*	Mean Square	*F-*value	*P-*value *P*rob > *F*
Linear	832.65	37	22.50	9.86	0.0021*
2FI	541.91	27	20.07	8.79	0.0032*
Quadratic	14.03	22	0.64	0.28	0.9899

Sequential Model Sum of Squares					
Source	Sum of Squares	*df*	Mean Square	*F-*value	*P-*value *P*rob > *F*
Linear vs Mean	445.36	5	89.07	4.62	0.0018*
2FI vs Linear	290.74	10	29.07	1.77	0.1044
Quadratic vs 2FI	527.88	5	105.58	102.03	< 0.0001*

Model Summary Statistics					
Source	Standard deviation	R-Squared	Adjusted R-Squared	Predicted R-Squared	PRESS
Linear	4.39	0.3442	0.2696	0.1588	1088.48
2FI	4.05	0.5689	0.3787	0.1495	1100.53
Quadratic	1.02	0.9768	0.9608	0.9474	68.01

“* Significant values, *df* : degree of freedom, PRESS: sum of squares of prediction error, two factors interaction: 2FI”.

The negative coefficient values (Tables 2, 3) suggest an antagonistic correlation among the variables, while the positive coefficient implies a synergistic relationship between the variables^40^. Consequently, the negative coefficients values of linear effects, mutual interactions and quadratic effects of the selected process parameters means that they exert a negative effect on Pb^2+^ and Congo red dye removal % by the dry biomass of *Ulva lactuca*, whereas the positive coefficient values mean that they increase Pb^2+^ and Congo red dye removal % by the dry biomass of *Ulva lactuca* in the tested ranges of the selected five process parameters. It's clear from the values of coefficients (Table 2) that the Pb^2+^ concentration and algal biomass concentration had positive effects on Congo red dye removal %. However, Pb^2+^ concentration, algal biomass concentration, initial pH level and contact time had positive effects on Pb^2+^ removal % (Table 3).

The Congo red dye regression model has R^2^ value = 0.9947 (Table 2), meaning that 99.47% of variations in the removal percentages of Congo red dye are attributed to the independent factors and the model cannot describe just 0.53 percent of the total variations. In addition, the Pb^2+^ regression model has R^2^ value = 0.9768 (Table 3), meaning that 97.68% of variations in the removal percentages of Pb^2+^ were due to the independent factors and the model cannot describe just 2.32 percent of the total variations. A regression model with a high R^2^ value greater than 0.9 and close to 1 considered to have the strongest, positive correlation and the model is good and would explain the variation of the experimental values as compared to the predicted values^41, 42^.

On the other hand, the values of the adjusted determination coefficient (Adj R^2^) are 0.9910 and 0.9608 for removal % data for both Congo red dye and Pb^2+^; respectively (Tables 2, 3). The very high values of Adj R^2^ verified that the model was very significant. The predicted R^2^ values of 0.9843 and 0.9474 for Congo red dye and Pb^2+^ removal %; respectively were in an excellent agreement with the adjusted R^2^ values that revealed a strong agreement between the predicted and observed values of Congo red dye and Pb^2+^ removal %. Therefore, the model used in this study is optimal in the range of experimental factors to predict Congo red dye and Pb^2+^ removal %.

In order to assess the significance of each parameter, *P*-values were used. In this case, the variables showing *P*-values below 0.05 were assumed to have significant effects^43^. The ANOVA of the regression model of response Y_1_ [percentage removal of Congo red dye] indicates that the model is highly significant as is apparent from a very small probability value [*P-*value ˂ 0.0001] with the calculated Fisher’s *F* test (*F-*value = 271.25) (Table 2). It was clear from the significance degree that the process variables linear coefficients including initial concentration of Congo red dye (X_1_), algal biomass concentration (X_3_), initial pH level (X_4_) and contact time (X_5_) were significant for Congo red dye removal with probability values of < 0.0001 (Table 2). The interaction effects between all factors are significant [*P-*value ˂ 0.05]. Furthermore, the *P*-values of the coefficients suggest that the quadratic effects of algal biomass concentration, initial pH level and contact time are significant. The quadratic effects of both initial concentrations of Congo red dye and Pb^2+^ are not significant (Table 2).

Similarly, the ANOVA of the regression model of response Y_2_ (Pb^2+^ removal %) indicates that the model is highly significant that is verified by a very small probability value [*P-*value ˂0.0001] with the calculated Fisher’s *F* test (*F-*value = 61.08) (Table 3). It was clear from the *P-*values that the linear coefficients of process variables including initial concentration of Pb^2+^, algal biomass concentration, initial pH level and contact time were significant for removal of Pb^2+^ with probability values of < 0.0001, 0.0005, < 0.0001, < 0.0001; respectively (Table 3). The interaction effects between all the variables are significant except interactions between X_1_X_4_ (Congo red dye concentration and initial pH level), X_2_X_3_ (Pb^2+^ concentration and algal biomass concentration) are not significant [*P-*value ˃ 0.05]. Furthermore, the *P*-values of the coefficients suggest that the quadratic effects of all the five variables are significant (Table 3).

The effect of initial Congo red concentration (X_1_) was not significant onto the removal percentage of Pb^2+^ and the effect of initial Pb^2+^ concentration (X_2_) was not significant onto the removal percentage of Congo red dye. Therefore, the percentages removals of Congo red dye and Pb^2+^ in the binary solution using the dry biomass of *Ulva lactuca* as biosorbent were not affected by the presence of each other. It could be assumed that Congo red dye and Pb^2+^ can be removed easily from binary solution utilizing the dry biomass of *Ulva lactuca* as biosorbent.

Statistically analyzed data of Congo red dye removal (%) shows that the value of coefficient of variation percentage is relatively low (C.V. = 3.03%) indicating that the experiments conducted have a high accuracy and reliability. Adequate precision determines the level of noise; the level higher than 4 is preferable and implies the model reliability. The present Congo red dye removal model had an acceptable adequate precision ratio of 63.80 and this means the reliability of the model. Value of PRESS for statistically analyzed data of Congo red dye removal (%) is 358.04. The model's mean and standard deviation values are 67.52 and 2.05; respectively (Table 2). At the same time, statistically analyzed data for Pb^2+^ removal (%) indicates that the coefficient of variation percentage (C.V. = 1.11%) is substantially small, suggesting that the experiments carried out have a high degree of precision and reliability The adequate precision ratio of Pb^2+^ removal model was 30.32 that imply the model reliability. The predicted residual square sum (PRESS) value is 68.01. The model's mean and standard deviation values are 91.48 and 1.02; respectively (Table 3).

Tables 4, 5 displays the fit summary results applied to determine the best polynomial model among linear, 2FI and quadratic models appropriate to the experimental data. The appropriate model was selected according to the significant model terms and an insignificant lack of fit test^40^. In addition, the model summary statistics indicate what model has higher adj. and pred. R^2^ and lower standard deviation. The fit summary results (Tables 4, 5) demonstrated that, the quadratic models of both Congo red dye and Pb^2+^ removal percentages by the *Ulva lactuca* dry biomass are very significant with a very small *P-*value < 0.0001. Lack of Fit Test for Congo red dye removal with *P-*value = 0.1062 and *F*-value = 2.52 is non-significant. The model summary statistics for Congo red dye removal quadratic model (Table 4) recorded the lower standard deviation of 2.05 and the highest adjusted R^2^ of 0.9910 and predicted R^2^ of 0.9843. Furthermore, Lack of Fit Test for Pb^2+^ removal with *F*-value = 0.28 and *P-*value = 0.9899 is non-significant. The appropriate model for Pb^2+^ removal was the quadratic model (Table 5), that would give predicted values close to the actual values for Pb^2+^ removal, with minimum residual values. The quadratic model recorded the highest adjusted R^2^ of 0.9608, predicted R^2^ of 0.9474 and the lowest standard deviation of 1.02.

The mathematical relationships between the chosen independent factors and the responses (Congo red dye and Pb^2+^ removal percentages) are given by the following polynomial regression equations of the second order using the coefficients determined:

1 $$\begin{gathered} {\mathbf{The}} \, {\mathbf{predicted}} \, {\mathbf{value}} \, {\mathbf{of}}\;{\mathbf{Congo}} \, {\mathbf{red}} \, {\mathbf{dye}} \, {\mathbf{removal}} \, \left( \% \right) = \, + {93}.{67} - {2}.{\text{77X}}_{{1}} + 0.{\text{58X}}_{{2}} + {5}.{\text{87X}}_{{3}} \hfill \\ - {6}.{\text{65X}}_{{4}} - {6}.{\text{57X}}_{{5}} - {2}.{\text{67X}}_{{1}} {\text{X}}_{{2}} - {1}.{\text{19X}}_{{1}} {\text{X}}_{{3}} + {3}.{\text{16 X}}_{{1}} {\text{X}}_{{4}} +_{{}} 0.{\text{95 X}}_{{1}} {\text{X}}_{{5}} + {3}.{\text{45X}}_{{2}} {\text{X}}_{{3}} {-}{4}.0{\text{3 X}}_{{2}} {\text{X}}_{{4}} \hfill \\ - {3}.{\text{29 X}}_{{2}} {\text{X}}_{{5}} + {4}.{\text{53 X}}_{{3}} {\text{X}}_{{4}} + { 3}.{\text{14 X}}_{{3}} {\text{X}}_{{5}} - {3}.{\text{71 X}}_{{4}} {\text{X}}_{{5}} + {2}.{\text{58X}}_{{1}}^{{2}} - {2}.{\text{43X}}_{{2}}^{{2}} - {3}.{\text{64X}}_{{3}}^{{2}} - {5}.0{\text{1X}}_{{4}}^{{2}} \hfill \\ - {29}.{\text{95X}}_{{5}}^{{2}} \hfill \\ \end{gathered}$$

2 $$\begin{gathered} {\mathbf{The}} \, {\mathbf{predicted}} \, {\mathbf{value}} \, {\mathbf{of}}\;{\mathbf{Pb}}^{{{\mathbf{2}} + }} {\mathbf{removal}}\left( \% \right) = \, + {96}.{49} - 0.{\text{26X}}_{{1}} + {2}.{\text{36X}}_{{2}} + 0.{\text{69X}}_{{3}} + {2}.{\text{16X}}_{{4}} \hfill \\ + {1}.{\text{53X}}_{{5}} + {1}.{\text{72X}}_{{1}} {\text{X}}_{{2}} - 00.{\text{99X}}_{{1}} {\text{X}}_{{3}} + 0.0{\text{7 X}}_{{1}} {\text{X}}_{{4}} +_{{}} {1}.{\text{46 X}}_{{1}} {\text{X}}_{{5}} + 0.0{\text{6X}}_{{2}} {\text{X}}_{{3}} + 0.{6}0{\text{ X}}_{{2}} {\text{X}}_{{4}} - {1}.0{\text{8 X}}_{{2}} {\text{X}}_{{5}} \hfill \\ + 0.{\text{68 X}}_{{3}} {\text{X}}_{{4}} - \, 0.{8}0{\text{ X}}_{{3}} {\text{X}}_{{5}} - 0.{\text{63 X}}_{{4}} {\text{X}}_{{5}} + {1}.{\text{47X}}_{{1}}^{{2}} - {1}.{\text{73X}}_{{2}}^{{2}} - {2}.{\text{99X}}_{{3}}^{{2}} - {2}.{\text{28X}}_{{4}}^{{2}} - {1}.{\text{83X}}_{{5}}^{{2}} \hfill \\ \end{gathered}$$

where X_1_–X_5_ are the coded levels of Congo red dye concentration, Pb^2+^ concentration, algal biomass concentration, initial pH level, and contact time; respectively.

### Three-dimensional surface plots (3D plots)

The three-dimensional surface plots were generated to show the relationships between the dependent variables (responses, Y_1_, Congo red dye removal percentages, Y_2_, Pb^2+^ removal percentages) and the interactions between the five selected independent variables, to assess the change of the response surface and to determine the optimal levels of selected process parameters for maximum removal of Pb^2+^ and Congo red dye from binary solution. 3D plots for the five variables (Congo red dye concentration, Pb^2+^ concentration, algal biomass concentration, initial pH level and contact time) combined in pairs were generated by plotting the Congo red dye removal (%) or Pb^2+^ removal (%) on Z-axis against two process parameters while other parameters were held fixed at their center points. Therefore, a total of ten three-dimensional surface plots were produced for each response [Y_1_, Congo red dye removal (%), Y_2_, Pb^2+^ removal (%)].

**Figure 2:**
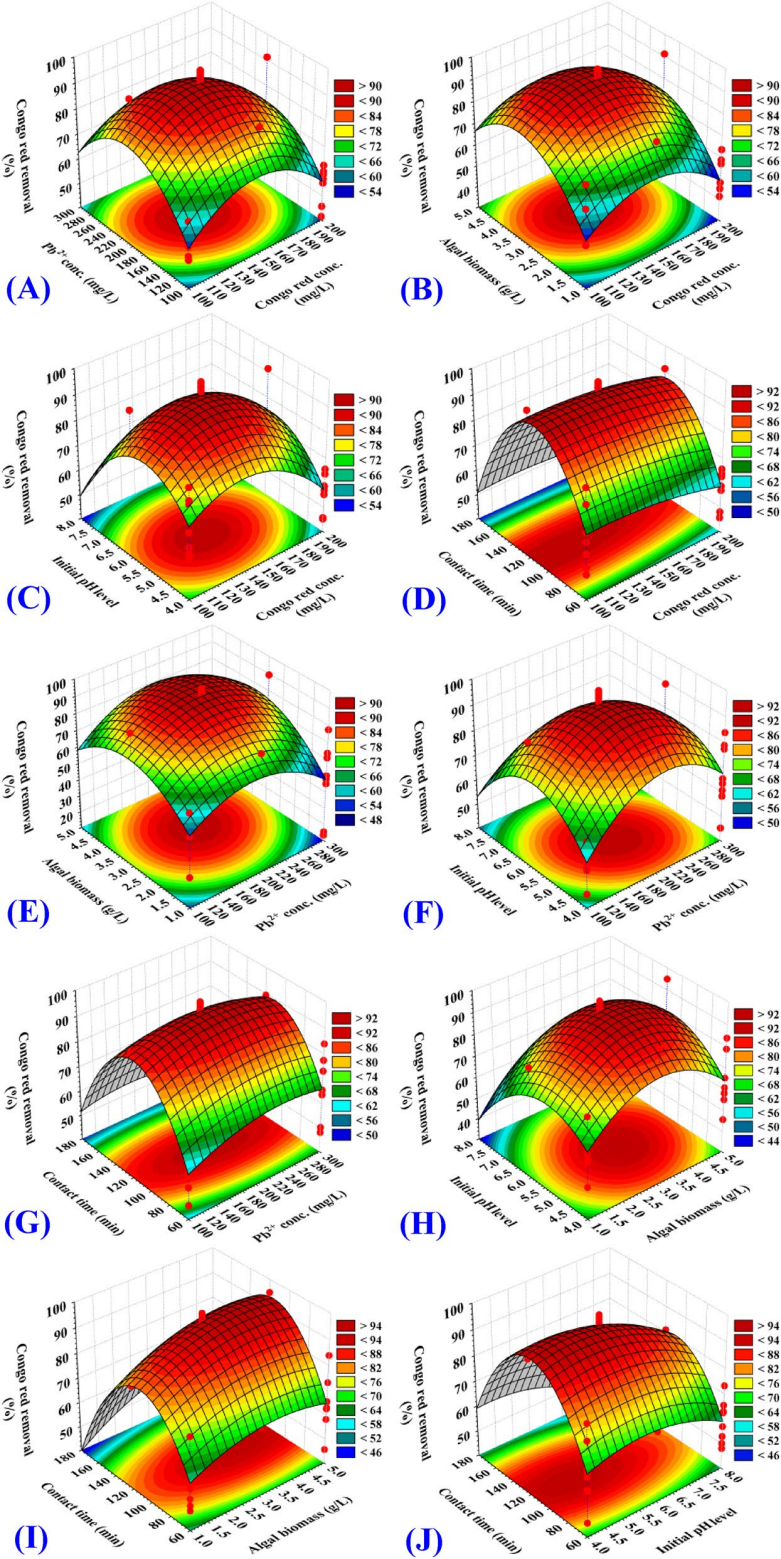
Three-dimensional surface plot for biosorption of Congo red by *Ulva lactuca* biomass, showing the interactive effects of the five tested variables.

**Figure 3:**
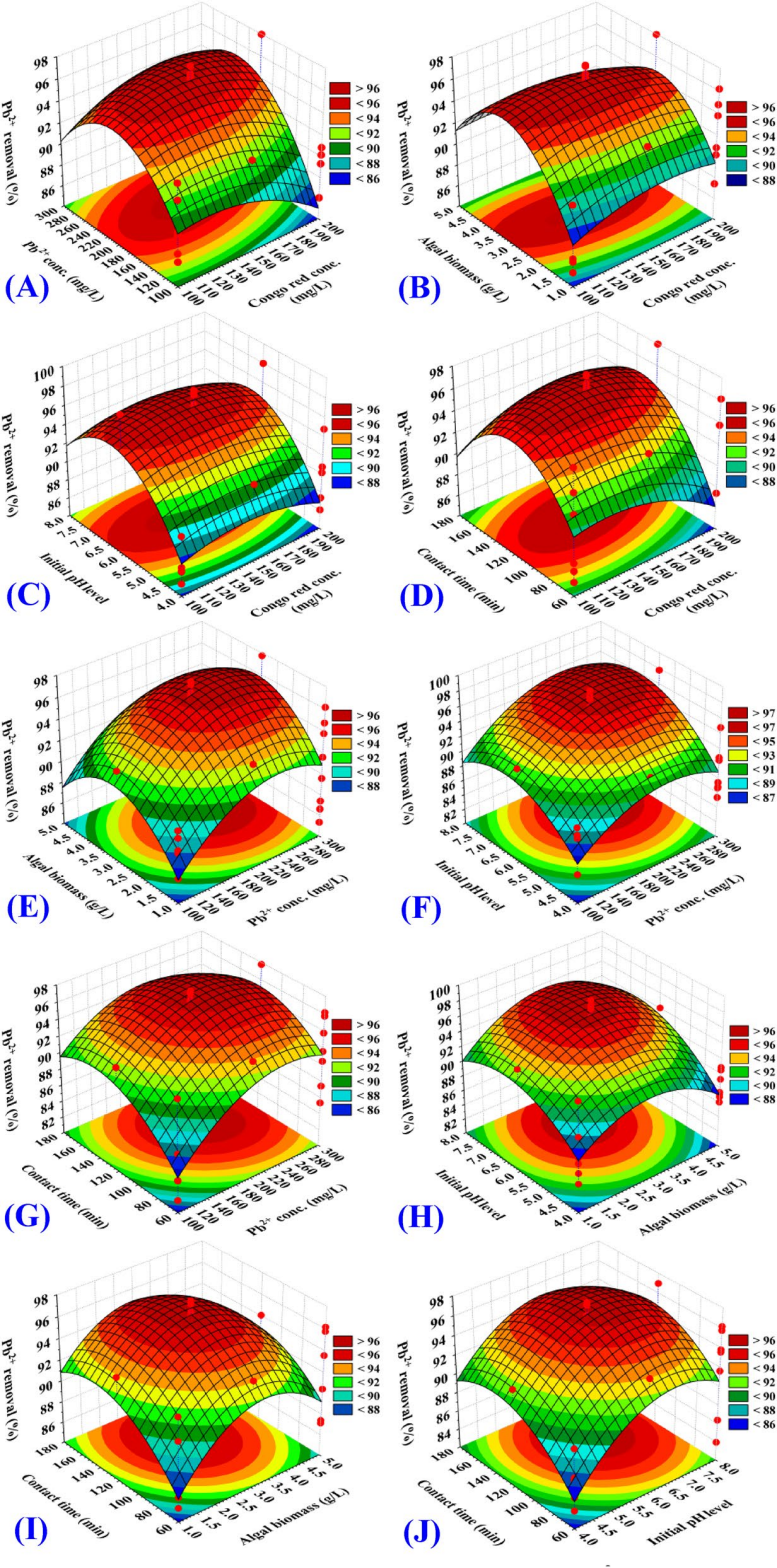
Three-dimensional surface plot for biosorption of Pb^2+^ by *Ulva lactuca* biomass, showing the interactive effects the five tested variables.

The three dimensional surface plots (Figs. 2A, 3A) show the simultaneous combined effects of initial Congo red dye concentration (X_1_) and initial Pb^2+^ concentration (X_2_) on Congo red dye removal efficiency (%) and Pb^2+^ removal efficiency (%), while algal biomass concentration, initial pH level and contact time (X_3_‒X_5_) were kept at their zero levels. In high and low initial concentrations of both Congo red dye and Pb^2+^, the percentage removal of Congo red dye (Fig. 2A) and Pb^2+^ (Fig. 3A) by *Ulva lactuca* biomass decreased, suggesting that the biosorption process was highly relied on both Congo red dye and Pb^2+^ initial concentrations.

### Effect of initial Congo red dye concentration on the biosorption process

The biosorption process of Congo red dye by *Ulva lactuca* biomass increased with increased concentration of Congo red dye. The maximum biosorption capacity with percent of 98.21% and 99.03% for Pb^2+^ and Congo red dye removal; respectively was obtained at the initial concentration of Congo red dye of 100.51 mg/L. The decrease in the removal percent of Congo red dye with further increases in its concentrations may be due to the depletion of available binding sites on the biomass of *Ulva lactuca* which were limited compared to an increasing number of dye molecules. The effect of dye concentration strongly influences the Congo red dye removal efficiency. Initial dye concentration provides a significant driving force to overcome any dye's mass transfer resistance between the aqueous and solid phase. A higher initial concentration of dye may enhance the adsorption process^44^.

### Effect of initial Pb^2+^ concentration on the biosorption process

The biosorption process of Pb^2+^ by *Ulva lactuca* biomass increased with the increase of the Pb^2+^ concentration. The maximum biosorption capacity was obtained at the initial Pb^2+^ concentration of 247 mg/L with percent of 98.13% and 100.01% for Pb^2+^ and Congo red dye removal; respectively. The amount of the biosorbed Pb^2+^ is influenced by the properties of *Ulva lactuca* biomass surface. An increase of the Pb^2+^ removal by increasing the initial Pb^2+^ concentration could be attributable to the increase in the driving force of the lead to overcome mass transfer resistance between the metal solution and the biosorbent^45^. The reduction in the removal percent of lead at higher concentrations could be attributable to the saturation of available binding sites on the *Ulva lactuca* biomass which was limited by increased competition for the fixed number of available binding sites in the biomass by increasing number of ions.

The three dimensional surface plots (Figs. 2B, 3B) show the simultaneous effect of Congo red dye concentration (X_1_) and algal biomass concentration (X_3_) on Congo red dye removal efficiency (%) and Pb^2+^ removal efficiency (%), while initial concentrations of Pb^2+^, initial pH level and contact time (X_2_, X_4_, X_5_) were held constant at their center points. The percentage removal of Congo red dye (Fig. 2B) and Pb^2+^ (Fig. 3B) increase with the increase in the initial algal biomass concentration. An increase of the Congo red dye and Pb^2+^ removal % by increasing the initial algal biomass concentration can be mainly due to the increased surface area and the number of unsaturated active sites available for the biosorption process reaction. The percentage removal of Congo red dye and Pb^2+^ then decreased with increasing the algal biomass concentration from 3.9 to 5 g/L. At high concentrations of algal biomass, the biomass agglomeration may be a reason for the decrease in the efficacy of removal.

Increase in the Congo red dye concentration resulted in increase in Congo red dye removal (%) up to 100.51 mg/L (by using 3.94 g/L of the algal biomass) and then further increase in the Congo red dye concentration decreased the removal percentage (Fig. 2B). Whereas, an increase in the concentration of Congo red dye has led to an increase in the percentage of Pb^2+^ removal up to 231.49 mg/L and then further increase in the Congo red dye concentration did not significantly affect the removal percentage of Pb^2+^ (Fig. 3B).

### Effect of biomass concentration on the biosorption process

Algae have proved to be effective sustainable biosorbents for metal removal owing to its low-cost and local abundance in both fresh and salt water and surface characteristics^30–32^. Schiewer and Volesky^46^ revealed high metal-binding capacities of algae attributed to the presence of lipid, proteins or polysaccharides in their cell walls containing functional groups like hydroxyl, amino and carboxylic that can act as binding sites for metals. There are many reports and reviews on the biosorption of lead ions on freshwater green algal species, green seaweed and marine algae^30, 47, 48^ with varying removal efficiencies. Each alga showed different metal-binding capacity. This could be explained with the difference in cell wall composition of polysaccharides and proteins which offers cell surface binding sites.

Dry biomass of *Ulva lactuca* was used as biosorbent for the simultaneous removal of Pb^2+^ and Congo red dye. At the lower biomass concentration, simultaneous biosorption of Pb^2+^ and Congo red dye was low. The simultaneous biosorption of both Pb^2+^ and Congo red dye from a binary mixture increases with increasing *Ulva lactuca* biomass concentration to 3.94 g/L. After reaching optimum biomass concentration, simultaneous biosorption of Pb^2+^ and Congo red dye by *Ulva lactuca* biomass was decreased.

Species of *Ulva* has extremely high surface area to volume ratio. Increase in the biosorption with increasing biomass concentration could be attributed to the increase in the surface area of *Ulva lactuca* biomass and the availability of more adsorption active sites. When the biomass is less, the active sites are effectively utilized. Phugare et al.^49^ reported that the increase in biosorption percentage with increasing biomass concentration is expected because of the increased surface area of the biosorbent that in turn increases the number of biosorption sites resulting in efficient biosorption. On the other hand, Karthikeyan et al.^50^ reported that a reduction in the effective surface area of biomass as a result of agglomeration may be a reason for a decrease in the efficacy of removal at high concentrations of algal biomass above 1 g. However, EL Hassouni et al.^51^ stated that the decrease in biosorption process efficiency with the increased biomass concentration can be attributed to the increased amount of unsaturated active adsorption sites on the biosorbent surface with the increased biomass concentration and insufficient available ions of the metals in the solution to binds with all the available binding sites. Garg et al.^52^ stated that the reduction in the removal percentage after the optimum dose was reached due to the filling of all active adsorption sites. As the concentration of biomass increased, the number of active adsorption sites increased and some of the available active sites may remain uncovered because of limited number of adsorbate molecules leading to lower specific uptake.

The three dimensional surface plots (Figs. 2C, 3C) show the simultaneous effect of Congo red dye concentration (X_1_) and initial pH level (X_4_) on Congo red dye removal efficiency (%) and Pb^2+^ removal efficiency (%), while initial concentrations of Pb^2+^, initial algal biomass concentrations and contact time (X_2_, X_3_, X_5_) were held constant at their center points. Increasing the initial solution pH resulted in an increase in the percentage of removal up to pH 6.51, and then a further increase in pH decreased the removal percentage. This means that the maximum removal percentages of Congo red dye and Pb^2+^ could be obtained by 3.94 g/L of the algal biomass and initial pH 6.51. The interaction effect between X_1_X_4_ (Congo red dye concentration and initial pH level) is significant for removal (%) of Congo red dye (*P-*value =  < 0.0001) (Table 2). While, as can be seen in Fig. 3C, the interaction effect between X_1_X_4_ is not significant (*P-*value = 0.7178) for removal (%) of Pb^2+^ by *Ulva lactuca* biomass (Table 3).

### Effect of initial pH value on the biosorption process

The initial pH of aqueous solution is the most important process factor not only affects the biosorption processes capacity of heavy metal ions from aqueous solution, but also the color and the solubility of some dyes. The simultaneous biosorption of Pb^2+^ and Congo red dye by *Ulva lactuca* biomass increases with a rise in pH and the maximum biosorption was reached around pH 6.5. At lower pH values (pH < 6.5), simultaneous biosorption of Pb^2+^ and Congo red dye by *Ulva lactuca* biomass was low, this could be explained by the higher concentration of the positively charged H^+^ ions (protons) on the surface active binding sites which compete with metal ions (cations). When the pH value increased to 6.5, the deprotonation of the binding sites makes different negative charges functional groups (such as carbonyle, carboxyl, amino and hydroxyl groups) available and the surface of the biomass was more negatively charged. With an increase in pH values, the formation of OH radicals has been increased^53^. Thus, the negatively charged functional groups facilitate the biosorption of metal ions that are positively charged.

The solution pH value has an influence on both solubility and degree of ionization of the metals in the solution and the ionization of biomass and the adsorption sites activities during the biosorption processes^54^. The net charge on marine algae is pH dependent and could influence the biosorption process due to their cell wall surfaces composed of polysaccharides^55^ which containing functional groups (adsorption sites) such as hydroxyl, amino, carboxyl and phosphates groups^56^. The ion exchange confirmed to be the dominant biosorption mechanism^57^. In another meaning, biomass can be considered as natural materials for ion exchange that mainly contain weak acidic and basic groups. At lower pH values of the metal solutions, binding sites in the biomass are protonated (positively charged) and repulsion occurs between the binding sites in the biomass and the heavy metal cations. Consequently, competition between protons (hydrogen ions) and heavy metals cations for binding sites decreases the heavy metals biosorption. At low pH, the H^+^ ions are high in concentration and can compete directly with heavy metal ions^55^. Other studies with seaweeds have indicated simultaneous release of H^+^ with the heavy metal ions biosorption. The Pb^2+^ biosorption occurs through the ion exchange mechanism in which, the Pb^2+^ binds to the binding sites at low pH by replacing two acidic H^+^. At higher solution pH values, the reduction in the process of biosorption is attributed to the formation of soluble hydroxylated complexes of the metal ions and their competition with the active sites, thus complicating the process of biosorption. The increased absorption of heavy metals has been due to decreased solubility and precipitation of metals^55^.

In this study, the optimum initial pH for maximum Congo red dye removal was 6.5 that similar to initial pH reported for Congo red removal by fungal biomass^58^. The Congo red dye is negatively charged and lead is a positively charged metal cations (lead can form 2^+^ or 4^+^ cations) (positively-charged ions), which neutralize their surface charges. At lower pH, the biosorbent surface becomes protonated and acquires net positive charge and thus, increases the binding of anionic dyes to the biosorbent surfaces. Higher pH values also increases the net negative charge on the biosorbent surface leading to electrostatic attraction of cationic dyes^59^. Vijayaraghavan and Shanthakumar^60^ reported that at low pH, strong electrostatic attraction between the anionic Congo red dye molecule and the alginate surface which is positively charged by absorbing H^+^ ions. Due to this electrostatic attraction, the Congo red dye molecules adhere on the positively charged alginate surface and settled down as sludge.

The three dimensional surface plots (Figs. 2D, 3D) show the simultaneous effect of Congo red dye concentration (X_1_) and contact time (X_5_) on Congo red dye removal (%) and Pb^2+^ removal (%), while keeping Pb^2+^ concentration, algal biomass concentration and initial pH level (X_2_, X_3_, X_4_) at their zero levels. The percentages removals of Congo red dye (Fig. 2D) and Pb^2+^ (Fig. 3D). Figures 2D, 3D show that the removal percentages of both Congo red dye and Pb^2+^ increased by increasing contact time. Then, further increase in the contact time above 102.94 to 180 min decreased the removal percentage of Congo red dye and Pb^2+^.

### Effect of contact time on the biosorption process

In the present study, the simultaneous removal of Pb^2+^ and Congo red dye by *Ulva lactuca* biomass from binary solution depends on the contact time. Experimental results have shown obviously that the percentage of Pb^2+^ and Congo red dye by *Ulva lactuca* biomass increases as the contact time increase up to the optimum, which may be due to the availability of vacant active sites on the *Ulva lactuca* biomass surface and also Pb^2+^ concentration is high. At higher contact time, the active adsorption sites of the *Ulva lactuca* biomass surface were saturated (occupied) causing no further adsorption occurs. Occupancy of all active adsorption sites causes saturation of biomass surface and results in a state of equilibrium^61^.

The percent biosorption of Congo red dye using the fresh water algae *Hydrilla verticillata* is rapid in the initial time because adequate surface area of the biosorbent is accessible for the biosorption of Congo red dye from aqueous solution. As time increase, the biosorption Congo red dye increased and the maximum percentage of biosorption is obtained after 25 minutes^62^. Mahajan and Kaushal^63^ reported that after 6 h of reaction time, maximum % of Congo red decolourization by *Chara vulgaris* was found in solution of initial dye concentrations of 10 ppm and minimum in initial dye concentrations of 40 ppm. However, after 24 h, 100% decolourization was obtained. The uptake capacity of Congo red dye by burned root of *Eichhornia crassipes* concentration was very fast at the initial contact time and at a higher contact time, it was slower until the equilibrium was reached after 45 min^64^. The rapid sorption during the initial contact time is probably due to the presence of a large number of surface active adsorption sites^64^. However, over time, it is difficult to occupy the remaining unsaturated surface active adsorption sites because of repulsive force between the Congo red dye molecules and bulk phases^64, 65^.

The surface plots (Figs. 2E, 3E) shows removal (%) of Pb^2+^ and Congo red dye as function of Pb^2+^ concentration (X_2_) and algal biomass concentration (X_3_) while other process parameters have been maintained at their zero levels. Congo red dye removal percentage (Fig. 2E) and Pb^2+^ removal percentage (Fig. 3E) increase with an increase in the initial concentration of algal biomass. The removal percentages of Congo red dye and Pb^2+^ decreased by using high and low algal biomass concentrations, indicating that the removal was highly dependent on algal biomass concentration. Increasing the initial Pb^2+^ concentration resulted in increased Congo red dye and Pb^2+^ removal and then further increase in the initial Pb^2+^ concentration decreased the removal percentage of Congo red dye (Fig. 2E). While, the rise of the initial concentration of Pb^2+^ up to 231.94 mg/L resulted in increase in the percentage removal of Pb^2+^ and further increase in the concentration of Pb^2+^ didn't affect the removal percentage of Pb^2+^ significantly (Fig. 3E). The interaction effect between X_2_X_3_ (Pb^2+^concentration and algal biomass concentrations) is significant for removal (%) of Congo red dye (*P-*value =  < 0.0001) (Table 2). While, the interaction effect between X_2_X_3_ is not significant (*P-*value = 0.7281) for removal (%) of Pb^2+^ by *Ulva lactuca* biomass (Table 3).

Figures 2F, 3F show that, by using low and high Pb^2+^ concentrations and initial pH level, the percentage of Pb^2+^ and Congo red dye decreased, suggesting that the removal was highly dependent on the initial pH level and Pb^2+^ concentration. Similarly, Figs. 2G, 3G reveals that increased contact time has contributed to an increase in both Congo red dye and Pb^2+^ removal percentages. From Figs. 2H, 3H, it is evident that by using low and high algal biomass concentrations and initial pH levels, the percentages of removal decreases. The effects of both algal biomass concentrations and contact time are also reflected in Figs. 2I, 3I. The effects of both initial pH level and contact time are also reflected in Figs. 2J, 3J.

### The model adequacy for analysis of Congo red dye removal (%)

**Figure 4:**
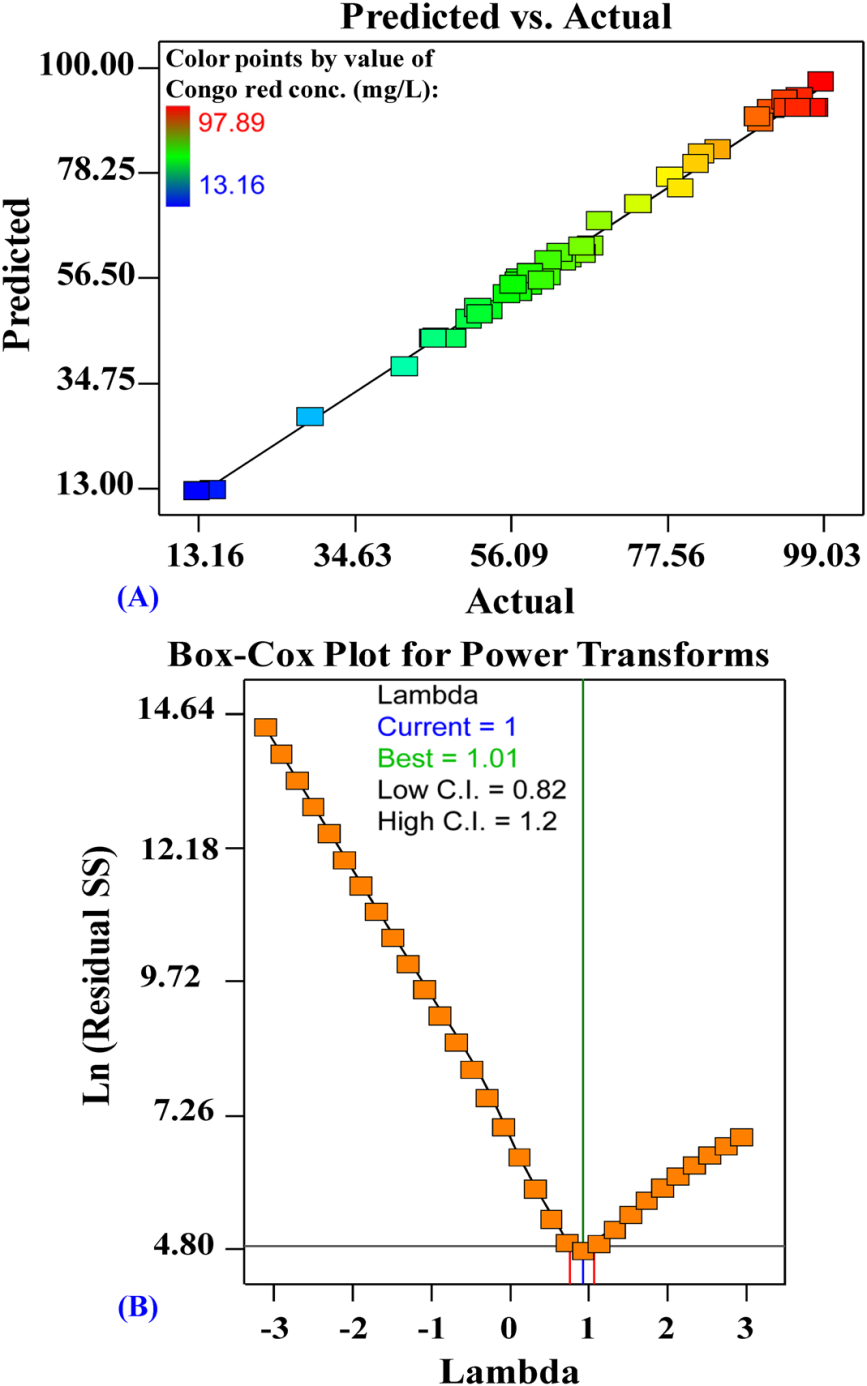
(**A**) plot of predicted versus actual, and (**B**) Box–Cox plot of model transformation of Congo red biosorption by *Ulva lactuca* biomass.

Figure 4A shows the actual versus predicted percentages for removal of Congo red dye from binary solution by *Ulva lactuca* biomass. Figure 4A displays all the points along the diagonal line, indicating that the model’s predicted percentages coincide with the actual percentages, confirming that the model is accurate. Figure 4B shows Box–Cox plot of the model transformation has generated for removal of Congo red dye from binary solution by *Ulva lactuca* biomass. Box–Cox plot of model transformation method can help checks data that are not normally distributed by transforming the data for normalization. As can be seen in Fig. 4B, the Lambda (λ) optimal value of 1 lies between the two vertical red lines so that no data transformation is required.

### The model adequacy for analysis of Pb^2+^ removal (%)

**Figure 5:**
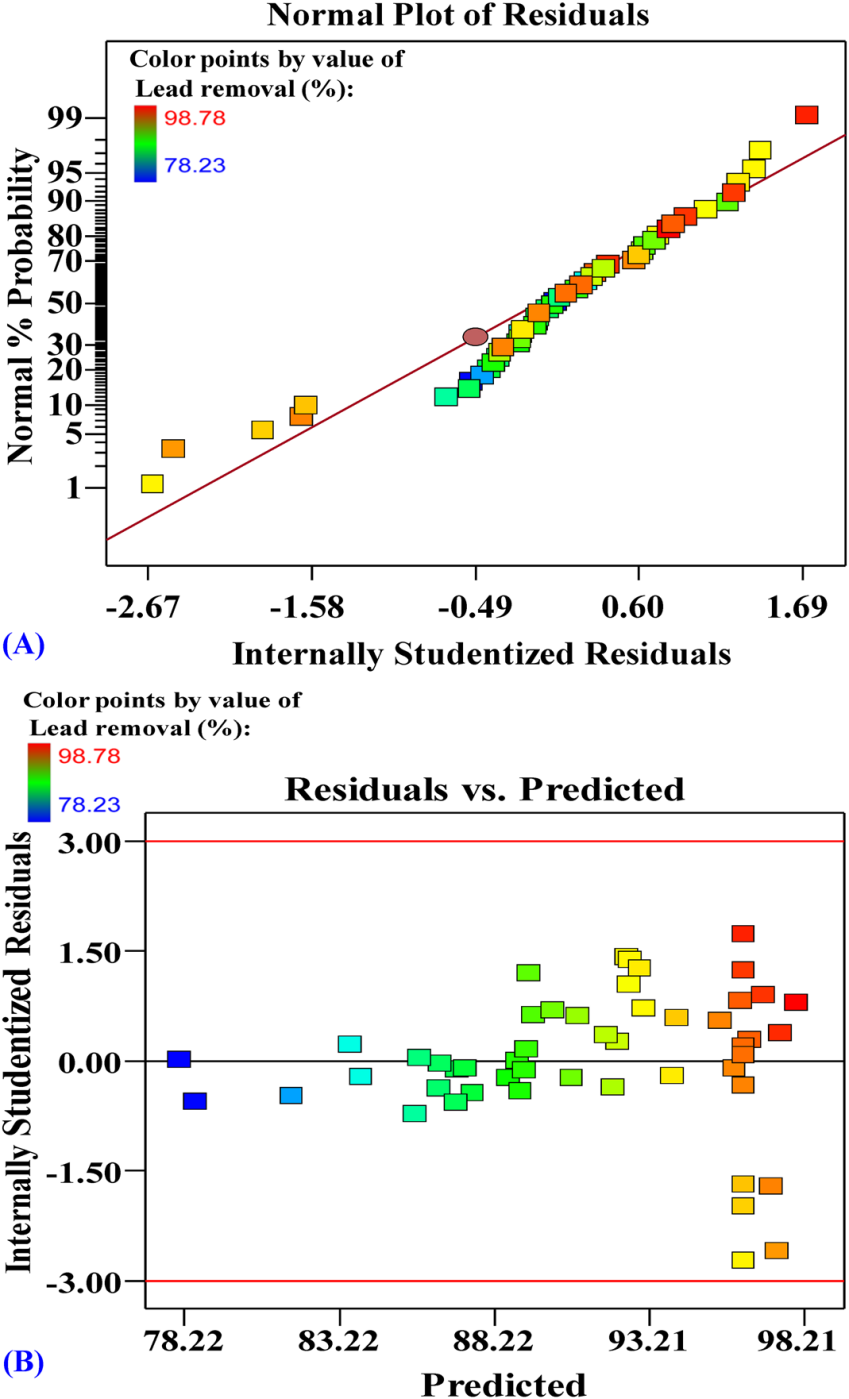
(**A**) Normal probability plot of internally studentized residuals, and (**B**) plot of internally studentized residuals versus predicted values of Pb^2+^ biosorption by *Ulva lactuca* biomass.

The normal probability plot (NPP) of the residuals is an effective graphical tool to verify the model suitability^38, 66^. Figure 5A shows the NPP of the residuals; the residuals are normally distributed along the diagonal line. This shows the adequacy of the model. A plot of predicted values vs. residuals of the removal (%) of Pb^2+^ (Fig. 5B) shows the points collected along the diagonal line indicating the adequate fit of the model.

### Desirability function (DF)

**Figure 6:**
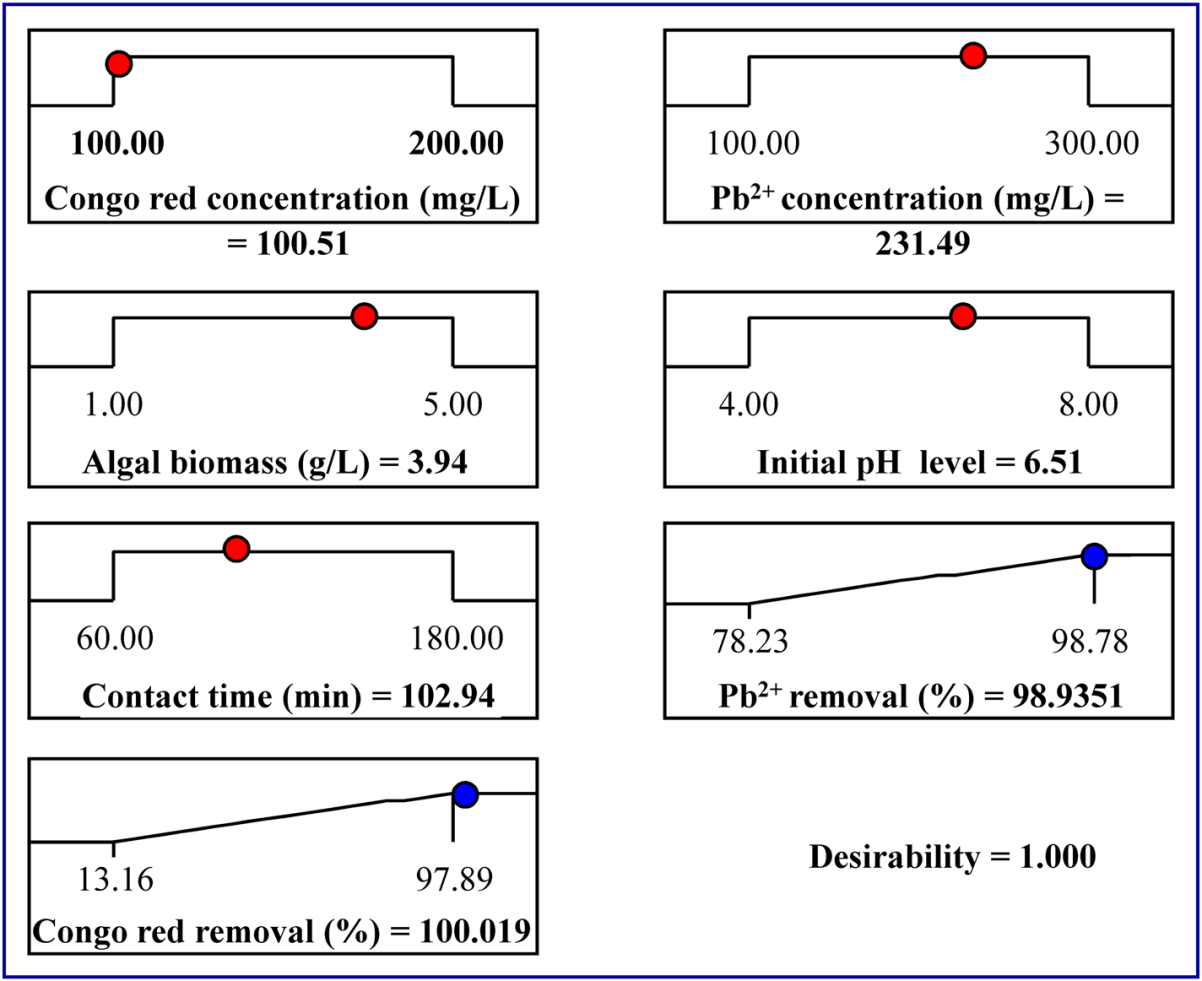
The optimization plot displays the desirability function and the optimum predicted values for the maximum percentage of Congo red dye and Pb^2+^ simultaneous biosorption.

The main goal of the experimental design is to determine the optimal predicted conditions for maximizing the responses. The desirability function (DF) was used to find the optimal predicted conditions for maximum response^67^. The values for DF varied from zero (undesirable) to one (desirable). The numerical optimization identifies the points which maximize the desirability function. The DF option in the Design Expert Software (version 7.0.0) was applied for the optimization process. The optimal predicted conditions attained using the desirability function for the maximum simultaneous removal of Congo red dye and Pb^2+^ by *Ulva lactuca* biomass (Fig. 6) were the initial Congo red dye concentration of 100.51 mg/L, initial Pb^2+^ concentration of 231.49 mg/L, the algal biomass concentration of 3.94, initial pH level of 6.51, and contact time of 102.94 min. The conditions resulted in the removal percentages of 98.94% and 100.01% (with DF of 1) for Pb^2+^ and Congo red dye; respectively.

In order to verify the removal percentages of Congo red dye and Pb^2+^ by *Ulva lactuca* biomass under the optimal predicted conditions, the experiments were performed in triplicate, and the experimental results compared with the predicted values. The removal percentages average of Congo red dye and Pb^2+^ were 99.1% and 98.65; respectively. It can be seen that the verification showed a high degree of agreement between the experimental and predicted values implies that the DF effectively determines the optimal predicted conditions for the simultaneous removal of Pb^2+^ and Congo red dye.

### Scanning electron microscopy (SEM) for morphology examination of the biomass surface of *Ulva lactuca*

**Figure 7:**
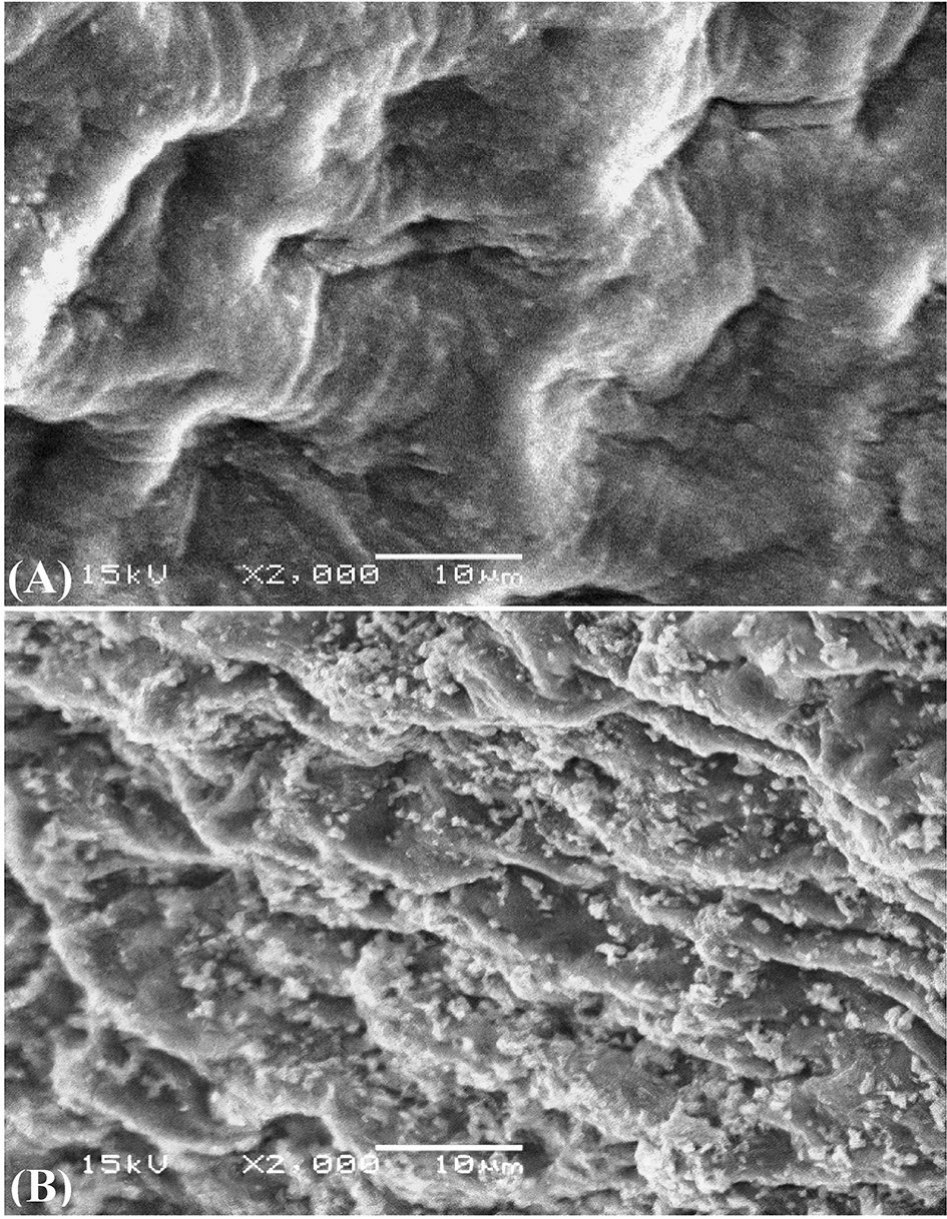
SEM micrograph of *Ulva lactuca* biomass: (**A**) before and (**B**) after simultaneous biosorption of Congo red and Pb^2+^.

The morphological features and surface characteristics of *Ulva lactuca* biomass were examined by SEM before and after the biosorption process. Figure 7A shows the micrograph of the surface of *Ulva lactuca* biomass before the biosorption process. It is revealed a regular surface of the *Ulva lactuca* biomass. Figure 7B shows the micrograph of the surface of *Ulva lactuca* biomass after biosorption of Congo red dye and Pb^2+^. Figure 7B revealed the presence of new glossy massive particles of the adsorbate ions on the surface of *Ulva lactuca* biomass which are absent on the surface of *Ulva lactuca* biomass before the biosorption process. Obvious morphological changes were seen in the cell surfaces, such as shrinking of surface. These changes may have been caused by vigorous cross-linking binding between Pb^2+^ and negatively charged functional groups in the polymers in their cell walls^68^.

### Fourier transform infrared (FTIR) spectra analysis

**Figure 8:**
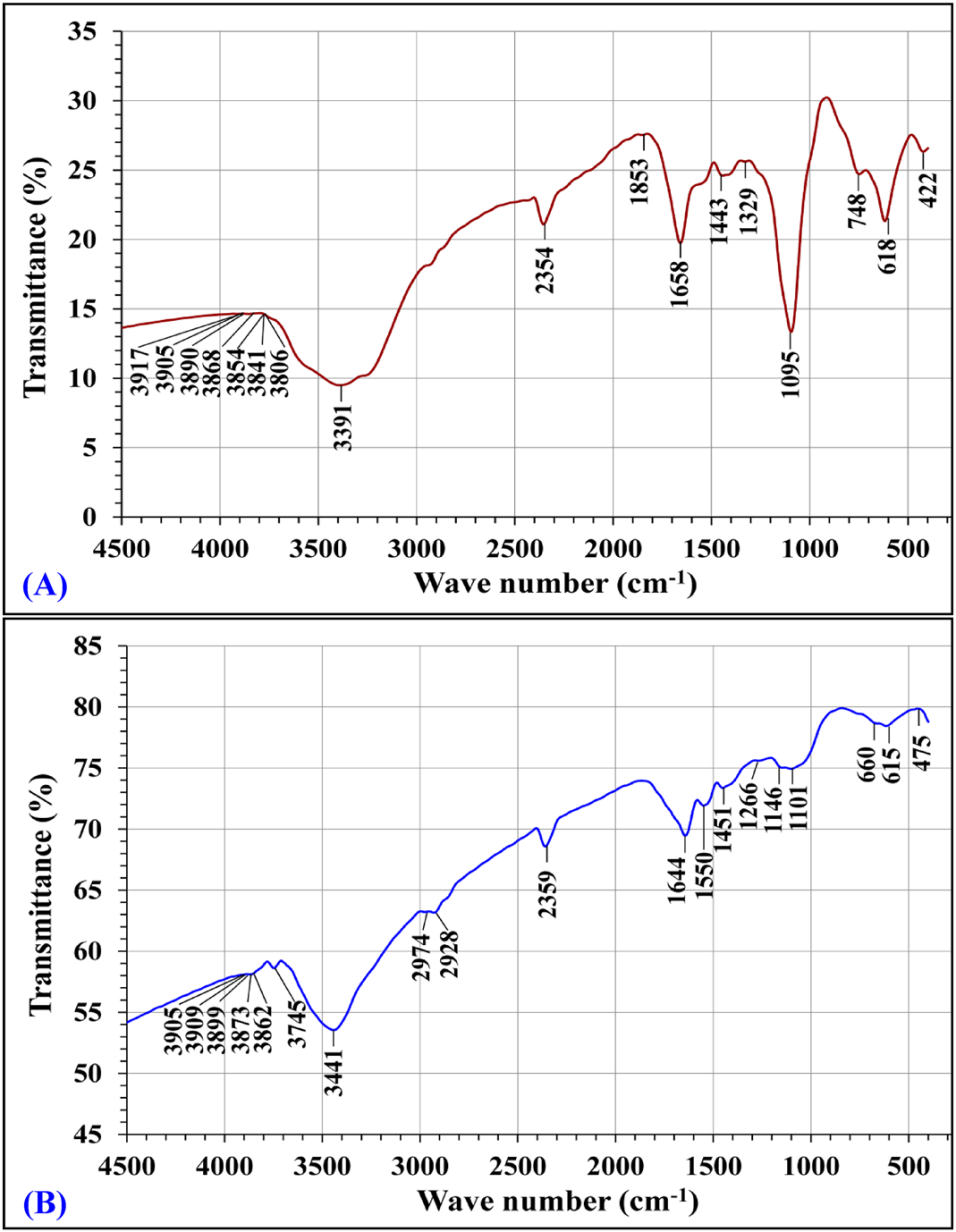
FTIR analysis of *Ulva lactuca* biomass: (**A**) before and (**B**) after simultaneous biosorption of Pb^2+^ and a carcinogenic Congo red dye.

FTIR spectroscopy was used to elucidate the chemical characteristics and to confirm the presence of functional groups on *Ulva lactuca* cell surface before and after biosorption of Congo red dye and Pb^2+^ to detect any differences in the surface characteristics. Each spectrum was collected within the wave number between 4000 and 400 cm^–1^ (Fig. 8). The degree of band shifting indicates the degree of interaction of functional groups with the adsorbate ions^69^. The main content of green algae biomass surface wall are proteins, which include functional groups such as carboxyl, amines, hydroxyl and sulphate^70^, which are responsible for the metal ions biosorption onto algal biomass through ion exchange and complexation reactions. Biosorption of metal ions takes place via the ion exchange process on the cell surface^71^. Moreover, these functional groups were deprotonated at pH value greater than their acidic dissociation constants and thus interacted with metal ions^72^. Vilar et al.^73^ have concluded that the biosorption of metal ions at pH < 7 is because of carboxylic groups (negatively charged) present in the biosorbents cell walls.

FTIR spectra (Fig. 8 and Table 6) for dry *Ulva lactuca* dry biomass sample before and after biosorption of Congo red dye and Pb^2+^. Significant shifts of peaks after the biosorption process indicates the degree of interaction and the significant role of the functional groups on the cell surface in the biosorption of Congo red dye and Pb^2+^. In the FTIR spectrum of the dry *Ulva lactuca* seaweed biomass sample before biosorption of Congo red dye and Pb^2+^, the characteristic vibrational band located at 3391 cm^–1^ is assigned to hydroxyl (–OH) groups^74^ is shifted to wave number at 3441 cm^–1^ which assigned to broad band of OH and NH^75^. Additionally, new two peaks have appeared in the FTIR spectrum of the *Ulva lactuca* biomass sample after the biosorption process of Congo red dye and Pb^2+^ at 2974 and 2928 cm^–1^. Vibrational band obtained at 2974 cm^–1^ is attributed to C–H group^76^, peak present at 2928 cm^–1^ is attributed to C-H stretching (–CH_3_ and –CH_2_ groups) which is specific to organic compounds such as glucose^77^. The vibrational FTIR peak located at 2354 cm^–1^ is corresponding to C–H stretching from aromatic group^78^ is transformed into the vibrational peak at 2359 cm^–1^ which is assigned to the N–H or C=O stretching vibrations^79^. In the FTIR spectrum of the dry *Ulva lactuca* seaweed biomass sample before biosorption of Congo red dye and Pb^2+^, the vibrational peak at 1853 cm^–1^ can be assigned to C–H stretching in trans HC=CH^80^, this peak disappeared in the FTIR spectrum for the biomass sample of *Ulva lactuca* after biosorption process. In parallel, the vibrational peak at 1658 cm^–1^ can be corresponding to NH_2_ bending, C=O, C=N stretching (amide I and II)^81^ is shifted to the vibrational peak at 1664 cm^–1^ of stretching vibrations of helical proteins, two neighboring peptide C=O groups^82^, the variational shift reveals that the amide groups are involved in the biosorption process through the ion exchange. On the other hand, new peak has appeared in the FTIR spectrum of the *Ulva lactuca* biomass sample after the Pb^2+^ and Congo red dye removal at 1550 cm^–1^ which corresponding to the presence of stretching vibrations of carbonates groups and C=C vibration^83^. The peaks at 1530–1560 cm^–1^ are characteristic for the amino groups (NH stretching)^69^. Additionally, Ata et al.^84^ indicated that the FTIR peaks located at 1640 and 1540 cm^−1^ indicate the presence of proteins and the IR absorbance peaks from 1660 to 1535 indicated possible assignmentof C=O, C=N stretching, NH_2_ bending (amide I and II)^81^. Sławomir^85^ reported that the FTIR peaks in the range of 1400 to 1657 cm^–1^ can be attributed to the stretching vibration of N–H bonds and C=O in the protein amide group. The vibrational absorption peak at 1443 cm^–1^ (before biosorption) represents C–H group stretching vibrations^86^ shifted to 1451 cm^–1^ (after biosorption process) which assigned to pure modes of CH_2_ scissoring vibrations^87^. The vibrational absorption peak appeared in the FTIR spectrum before biosorption process at 1329 cm^–1^ can be corresponding to N–H group^88^. A vibrational shift in the FTIR peak to lower wave number at 1266 cm^–1^ (after biosorption process) indicated alterations in NH bending (amide III), O–H stretching, carboxylic groups and C=O functional group of the oligo and polysaccharides^89^. The major peak shift (–63) suggests the association of amide, phenolic and carbonyl groups in the biosorption process. Before biosorption, the major vibrational band at 1095 cm^–1^ represents stretching vibrations of C–O^90^ is transformed after biosorption process into two minor vibrational peaks at 1101 and 1146 cm^–1^ which assigned to C–O–C and C–O ring^91^. The presence of vibrational peaks in the range of 1000 to1100 cm^−1^ is characteristic of polysaccharides^84^. The vibrational absorption peak appeared in the FTIR spectrum before biosorption process at 748 cm^–1^ can be attributed to C–H wagging^92^, a vibrational shift in this peak to lower wave number at 660 cm^–1^ (after biosorption process) indicated symmetric deformation of an ionized carboxylic group (COO) stretching vibration^93^. The major peak shift (–88) suggests that the carboxylic groups were involved in the biosorption process. However, the vibrational peak appeared in the FTIR spectrum before biosorption process at 618 cm^–1^ attributed to C–C twisting (protein)^94^ is shifted to the vibrational peak at 615 cm^–1^ that is characteristic of C–H group, sulfates (S–O bend vibration)^95^. The low frequency vibrational peak appeared in the FTIR spectrum before biosorption process at 422 cm^–1^ is characteristic of O–H group^96^ or C=N^97^ is shifted to high frequency absorption band at 475 cm^–1^ which is either due to harmonics of O=P–O linkages, bending vibration of O–P–O^98^ or S–S band^99^.

**Table 6:** FTIR spectral analysis of *Ulva lactuca* biomass before and after biosorption of Congo red and Pb^2+^.

Before biosorption		After biosorption		Shift	References
Wave no. (cm^−1^)	Annotations	Wave no. (cm^−1^)	Annotations		
3917 3905 3890 3868 3854 3841 3806	O–H stretching	3959 3909 3899 3873 3862 3754	O–H stretching	+ 42 + 4 + 9 + 5 + 8 –87	Andrews and Hunt^73^
3391	O–H hydroxyl group	3441	broad band of OH and NH	+ 50	Widjanarko et al.^74^, El-Sheekh et al*.*^75^
–	–	2974	C–H	–	Khan et al. (2008)^76^
–	–	2928	C–H	–	Ouhaddouch et al*.*^77^
2354	C–H stretching from aromatic group	2359	N–H or C=O stretching vibrations	+ 5	Maaz^78^, Raman^79^
1853	C–H stretching in trans HC=CH		–	–	Sun^80^
1658	C=O, C=N stretching, NH_2_ bending (amide I and II)	1644	C=O Carbonil	–14	Filip and Hermann^81^, Byler and Susi^82^
–	–	1550	Carbonates groups	–	Wilsona et al*.*^83^
1443	C–H group (alkyl)	1451	Pure modes of CH_2_	+ 8	Prosanov and Matvienko^86^, Pawlukojć et al.^87^
1329	N–H group	1266	O–H stretching, NH bending (amide III), C=O, carboxylic groups	–63	Greenwood^88^, Kumari et al*.*^89^
1095	C–O stretching	1146 1101	C–O–C C–O ring	+ 51 + 6	Shao and Wei^90^, Kačuráková et al*.*^91^
748	C–H wagging vibration	660	Carboxylic group (COO–) stretching vibration	–88	Da Róz et al.^92^, Ramaswamy et al.^93^
618	C–C twisting (protein) (alkanes)	615	C–H group, S–O bends inorganic sulfates	–3	Rehman et al.^94^, Smidt and Meissl^95^
422	C=N or O–H stretching	475	S–S band	+ 53	Kong and Shaoning^97^, Carpenter et al.^99^

These shifts in the absorption peaks confirm the association of these functional groups on *Ulva lactuca* biomass in the biosorption of Congo red dye and Pb^2+^. In conclusion, FITR spectra confirmed that the methyl, alkanes, amide, phenolic (hydroxyl), carbonyl, alkyl, carboxylic, sulfates and phosphate groups were the principle groups involved in the Congo red dye and Pb^2+^ biosorption process.

### Electron dispersive spectroscopy (EDS)

**Figure 9:**
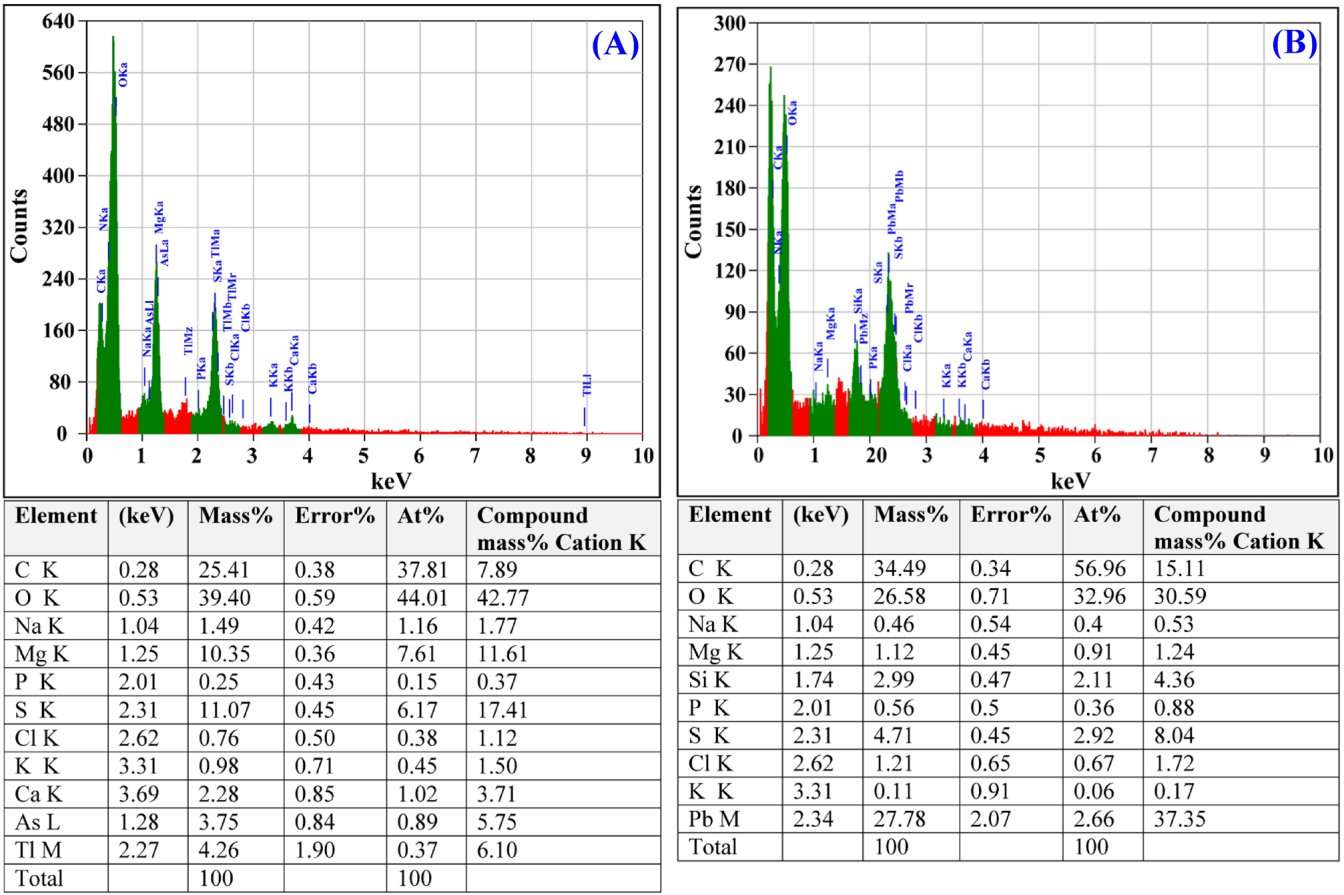
EDS analysis of *Ulva lactuca* biomass: (**A**) before and (**B**) after simultaneous biosorption of Congo red and Pb^2+^.

Energy-dispersive spectroscopy (EDS) is a valuable tool for chemical characterization or elementary analysis of biosorbents^100^. In the present study, EDS analysis was conducted to confirm the presence of Pb^2+^ attached to *Ulva lactuca* cell surface. After the biosorption process, the EDS spectrum (Fig. 9B) reveals the presence of additional peak of Pb^2+^ (Fig. 9A) confirming the capacity of *Ulva lactuca* biomass for removal of Pb^2+^ from binary solution.

## Materials and methods

### Collection and preparation of the biosorbent

*Ulva lactuca* used in this study was harvested from the Mediterranean Sea coast of Abu-Qir, Alexandria, Egypt during summer season, 2019. The alga was thoroughly washed with fresh running tap water to remove salts, sand and any other external impurities. The washed algal biomass was then dried at room temperature until a constant weight was obtained. The dried algal biomass was ground and the obtained fine particles with an average size of 0.3‒0.5 mm were selected and stored in dry place for further use.

### Preparations of Congo red dye and lead stock solutions

The Congo red dye stock solution was prepared by the precise dissolving of weighted Congo red dye for a concentration of 1000 mg/L in distilled water. The desired work concentrations were then prepared by dilution of Congo red dye stock solution.

Lead stock solution was prepared by the precise dissolving of weighed lead nitrate (Pb(NO_3_)_2_) in distilled water at a concentration of 1000 mg/L. The desired work concentrations were then prepared by dilution of stock solution of lead.

### The biosorption experiments

The biosorption experiments were conducted in batch mode in 250 mL flasks with 100 mL working volume to study the effect of lead concentration, initial pH, contact time, Congo red concentration and algal biomass concentration on the biosorption process. Dry *Ulva lactuca* biomass was thoroughly mixed with the previously prepared solutions of Congo red and Pb^2+^ with different concentrations as demonstrated in the 50 trials (Table 1). The initial pH has been adjusted with the addition of 0.1 N NaOH or 0.1 N HCl to each solution^30^. The suspensions were incubated and agitated at 150 rpm in a shaker incubator at 30ºC for a period contact time.

### Optimization of biosorption experiments for simultaneous biosorption of Pb^2+^ and a Congo red dye by Face- centered central composite design (FCCCD)

FCCCD was used to determine the optimum levels of five factors (lead concentration, pH, contact time, Congo red concentration and biosorbent (algal biomass) concentration) to achieve the maximum efficiency for simultaneous bioremoval of Congo red dye and Pb^2+^ from binary solution. In addition, to study the linear, quadratic and mutual interactions effects among the selected process factors that have a significant impact on the biosorption process. FCCCD of 50 experimental runs in a random order, 10 axial points, 32 factorial and 8 replicates at the midpoints were used. The five factors vary on three coded levels (− 1, 0 and + 1) (Table 1). The responses values (Y) for Congo red dye removal (%) and Pb^2+^ removal (%) in each trial were the average of the triplicate. All experiments were performed at 30 °C to save energy.

The correlations between the selected tested factors and the responses (Pb^2+^ and Congo red dye biosorption percentages) were determined using the equation of second-degree polynomial as follows:

3 $$Y = \beta_{0} + \sum\limits_{i} {\beta_{i} X_{i} + \sum\limits_{ii} {\beta_{ii} X_{i}^{2} } } + \sum\limits_{ij} {\beta_{ij} X_{i} X_{j} }$$

In which Y is the predicted Pb^2+^ or Congo red dye biosorption, β_ij_ (interaction coefficients) , β_ii_ (quadratic coefficients), β_i_ (linear coefficient), β_0_ (regression coefficients) and X_i_ is the tested factors coded levels.

### Statistical analysis

STATISTICA and Design Expert version 7 for Windows softwares were used for the experimental designs, statistical analysis and to draw the plots of three-dimensional surface.

### Analytical methods

The content of each flask for FCCCD experiment was centrifuged at 6000 × *g* and analyzed using Atomic Absorption spectroscopy according to “standard methods for the examination of water and wastewater 23rd edition 2017”^101^. The efficiency of *Ulva lactuca* biomass as biosorbent for lead ion removal was quantitatively calculated as follows:

4 $${\text{Removal efficiency (\%) = }}\frac{{{\text{C}}_{{\text{i}}} - {\text{C}}_{{\text{f}}} }}{{{\text{C}}_{{\text{i}}} }} \, \times 100$$

where: Where: C_i_, C_f_ are the initial and final concentrations of lead ions (mg/L); respectively.

The residual Congo red concentrations were determined spectrophotometrically on a UV/Vis spectrophotometer at wavelength of the highest absorbance (λ_max_) that was 500. Congo red removal % was determined as follows:

5 $${\text{Removal efficiency (\%) = }}\frac{{{\text{C}}_{{\text{i}}} - {\text{C}}_{{\text{f}}} }}{{{\text{C}}_{{\text{i}}} }} \, \times 100$$

where: C_i_ and C_f_ are the initial and residual concentrations of Congo red dye (mg/L); respectively.

### Material characterization

The morphological features and surface characteristics of *Ulva lactuca* biomass were examined before and after biosorption of Congo red dye and Pb^2+^ using FTIR spectra analysis, Scanning Electron Microscopy (SEM) and Energy-dispersive spectroscopy (EDS) analysis.

### Fourier-transform infrared (FTIR) spectroscopy

FTIR analyses were performed in order to elucidate the distinctive surface chemical characteristics (functional groups) of the dehydrated *Ulva lactuca* biomass samples that may be relevant to binding of Pb^2+^ and Congo red dye to the biomass. The samples of *Ulva lactuca* biomass were analyzed before and after Pb^2+^ and Congo red dye biosorption with the FTIR spectroscopy (Thermo Fisher Nicolete IS10, USA spectrophotometer). The dry algal biomass samples of *Ulva lactuca* were mixed with pellets of potassium bromide. The FTIR spectra were analyzed within the range of 400–4000 cm^−1^.

### Cell wall surface analysis by scanning electron microscopy (SEM)

The dehydrated *Ulva lactuca* biomass samples were coated with gold and examined before and after biosorption of Pb^2+^ and Congo red dye with SEM to investigate the algal cell surface and to assess the biosorption of Pb^2+^ and Congo red dye by the biomass.

### Energy-dispersive spectroscopy (EDS) analysis

The dehydrated *Ulva lactuca* biomass samples before and after biosorption of Pb^2+^ and Congo red dye were examined using SEM/EDS to determine the chemical composition.
